# Morphometrical and Immunohistochemical Evaluation of Kidney as an Indirect Parameter to Estimate Age in Puppies in Veterinary Forensic Pathology

**DOI:** 10.3390/ani13162665

**Published:** 2023-08-18

**Authors:** Ilaria d’Aquino, Giuseppe Piegari, Gianluca Miletti, Emanuela Sannino, Dario Costanza, Leonardo Meomartino, Rosario Fico, Lorenzo Riccio, Emanuela Vaccaro, Davide De Biase, Orlando Paciello

**Affiliations:** 1 Unit of Pathology, Department of Veterinary Medicine and Animal Production, University of Naples Federico II, 80137 Naples, Italy; ilaria.daquino@unina.it (I.d.); dario.costanza@unina.it (D.C.); leonardo.meomartino@unina.it (L.M.); lorenzo.riccio@unina.it (L.R.); emanuela.vaccaro@unina.it (E.V.); paciello@unina.it (O.P.); 2 Unit of Forensic Veterinary Medicine, Department of Animal Health, Istituto Zooprofilattico Sperimentale del Mezzogiorno, 80055 Naples, Italy; gianluca.miletti@izsmportici.it (G.M.); emanuela.sannino@izsmportici.it (E.S.); 3 National Reference Centre for Veterinary Forensic Medicine, Istituto Zooprofilattico Sperimentale delle Regioni Lazio e Toscana, 58100 Grosseto, Italy; errefico@gmail.com; 4 Department of Pharmacy, University of Salerno, 84084 Salerno, Italy; ddebiase@unisa.it

**Keywords:** puppies, forensic, dog, kidney, age estimation

## Abstract

**Simple Summary:**

Estimation of age represents a challenge in the veterinary forensic pathology field as well as in human forensic medicine. In puppies, the most common methods of age estimation are the visual examination of the dentition and the skeletal age. Nevertheless, these methods are affected by a broad range of variables. In contrast, the kidney is characterized by a precise postnatal development. In human glomerulogenesis, fetal mesangial cells change their immunohistochemical phenotypes with maturation. Consequently, we hypothesized that histological and immunohistochemical evaluations of the kidney can be used together as an indirect parameter for age estimation in puppies’ cadavers. Kidney samples from 45 cadavers were collected and processed for histopathological (for morphometrical study of the glomerulus) and immunohistochemical (for the immunolocalization of the α-smooth muscle actin protein—α-SMA) examinations. Morphometrical and immunohistochemical studies allowed us to observe differences among assessed groups. Our findings suggest a potential use of both kidney morphometrical and immunohistochemical examinations together as an indirect parameter to assess the age of puppies.

**Abstract:**

Estimation of age represents a central focus in the veterinary forensic pathology field. Currently, the visual examination of the dentition and the skeletal age are the main methods to estimate the age of puppies. Nevertheless, these methods are affected by a broad range of variables. In contrast, the kidney is characterized by a specific postnatal development. In human glomerulogenesis, fetal mesangial cells change their immunohistochemical phenotypes with maturation. Therefore, we hypothesized that histological and immunohistochemical examinations of the kidney can be used together as an indirect parameter for age determination in puppies’ cadavers. Forty-five puppies’ cadavers were divided into five groups defined by age (Group A= 0–15 days, Group B = 16–45 days, Group C = 46–85 days, Group D = 86–105 days, Group E= 105–365 days). For each case, kidney samples were collected and processed for histopathological (for morphometrical study of the glomerulus) and immunohistochemical (for the immunolocalization of the α-SMA protein) studies. Morphometrical study allowed us to observe statistical differences in the mean glomerulus numbers per field among assessed groups. Similarly, immunohistochemical examination showed differences in SMA expression among groups. Our findings suggest a potential use of kidney morphometrical and immunohistochemical examinations together as an indirect parameter to assess the age of illegally imported puppies.

## 1. Introduction

Assessment of age represents a central focus in veterinary forensic pathology as well as in human forensic medicine. Age estimation of children and adolescents is a subject of fundamental importance in the medico-legal field; it is required in order to corroborate if there is a legal responsibility, juridical implication, or in cases of adoption processes and asylum requests. There are several anatomical and morphological regions that can be used to establish the age of an individual; they are defined as “age indicators” and are represented by sexual features, teeth, and bones. Therefore, a wide number of age estimation techniques have been proposed in living human beings but only few of them are suitable for forensic application [1]. The most common methods used are the radiographic evaluation of the carpal/hand area (used for age estimation of children and adolescents) [2] and the third molar index method (used to recognize subjects who have survived eighteen years of age) [3,4,5]. Accuracy with which the age of adolescents can be estimated, and the factors that influence it, have been widely evaluated in human literature. In contrast, in veterinary literature, few reports have investigated the methods to estimate the age of dogs. However, in recent years, age determination has assumed significant relevance for correctly determining the age of puppies illegally imported into European countries as well as puppies that are sold by breeders earlier than 8 weeks of age. In some circumstances, puppies are precociously separated from their mothers and littermates and transported illegally within the European Community [6,7]. In order to be moved within the European Union, puppies must be vaccinated against rabies [8,9]; the vaccine must be administered no earlier than 12 weeks of age and the period of efficacy of the vaccination starts not less than 21 days from the completion of the vaccination protocol. Therefore, the minimum age for puppies’ movements in the European Union is 105 days old. Furthermore, behavioral consequences of dogs prematurely separated from their mothers and littermates has been extensively studied [10,11]; in particular, separating a puppy from its mother during this time, especially during the early stages, can be a traumatic experience. Such trauma can result in stress, anxiety, aggression, distrust of strangers, and difficult behavior with other dogs. Moreover, research has shown a correlation between early mother–puppy separation and behavioral alterations in adults, including being fearful, aggressive, anxious, and being more difficult to train [12,13]. For these reasons, veterinarians are increasingly demanded to precisely estimate the age of puppies. In veterinary literature [14,15,16], the main methods to evaluate the age of living puppies are the visual examination of the dentition, specifically the completeness of dental eruption and the extent of tooth wear [17,18,19,20,21,22,23,24]. Another technique based on teeth examination is counting dental cementum annuli [25,26,27,28,29,30]; cementum deposition starts at about the time of tooth emergence and, in mammals, there is the appearance of one annulus every year of the animal’s life. In dogs, to the best of our knowledge, this method has never been investigated, but it is described that in wild gray wolves the first annulus deposition occurs between 18 and 22 months of age [28]. For these reasons, this technique could not be used in animals younger than 18 months old. Another age estimation method used in mammals is the evaluation of the skeletal age, specifically the radiographic appearance and formation of ossification centers in fore- and hind-limbs bones [31,32,33,34,35,36,37]. Moreover, these methods are affected by environmental factors, such as nutritional, hormonal, and pathological changes [14,18,38,39]. Previous studies in human glomerulogenesis showed that fetal mesangial and endothelial cells change their immunohistochemical expression of specific proteins, such as alpha smooth muscle actin (α-SMA), with maturation [40]. α-SMA is one of the six isoforms of the intra-cellular microfilament actin and it is usually considered to be a marker for smooth muscle-derived tissue. Furthermore, it can also be expressed in non-muscle cells; in fact, α-SMA expression has been previously described in the kidney glomerulus in fetuses and infants [40] and in pericytes of the renal medulla [41]. In addition, the kidney is characterized by a precise postnatal development in which this organ continues to mature both from a functional and anatomical point of view with a progressive maturation of glomeruli in the nephrogenic zone [42]. Therefore, our hypothesis was that the different postnatal stages of renal development may be used together as an indirect parameter for age determination in puppies’ cadavers. Considering these observations, the aim of this study was to investigate the correlation between age and morphological and immunohistochemical (alfa-smooth muscle actin) modification of glomeruli in puppies’ cadavers.

## 2. Materials and Methods

### 2.1. Study Design

Forty-eight dead puppy dogs (medium-sized animals, age range 1–365 days) were enrolled in the study. None of the studied dogs showed any clinical evidence of renal disease. Each owner consented to use of the cadaver for research purposes, according to the ethical guidelines of the Department of Veterinary Medicine and Animals Production of the University of Naples Federico II. Animals were divided into five groups as follows: Group A comprised twenty cadavers of dogs with an age between 1 and 15 days; Group B comprised seven cadavers with an age between 16 and 45 days; Group C included eight cadavers with an age between 46 and 75 days; Group D comprised seven cadavers with an age between 76 and 105 days. Finally, the Group E comprised six cadavers of animals with an age between 105 and 365 days.

### 2.2. Macroscopic and Histological Examinations

Forensic necropsies were conducted in all cadavers in the necropsy room of the Department of Veterinary Medicine and Animal Production of the University of Naples “Federico II” and in the necropsy room of the Istituto Zooprofilattico Sperimentale del Mezzogiorno (Portici) following the standard forensic necropsy protocol [43]. Cadavers showed different degrees of postmortem autolysis. For each case, 1 cm thick kidney samples were collected for histopathologic examination; samples were rapidly transferred and fixed in 10% neutral buffered formalin (05-01005Q, BioOptica, Milan, Italy) for 7 days. After fixation, sections were trimmed, placed into the cassette, dehydrated, and embedded in paraffin wax. Kidney specimens were sectioned with a microtome (Rotary 3003, pfm, BioOptica, Milan, Italy) into 4 μm thick sections, mounted on a slide and stained with hematoxylin and eosin (H&E) (Leica ST5010 Autostainer XL). A histological and morphometrical study was performed to determine if there was a correlation between the number of glomeruli and the age of puppies. The degree of tissue autolysis was evaluated according to the postmortal interval. Postmortem kidney histological changes were scored as follows: “Mild” (1): minimal changes, normal cytoplasm, and nuclei of cells visible with identifiable glomeruli; “Moderate” (2): loss of cells details but with nuclei and cellular outline still visible and identifiable glomeruli; “Severe” (3): loss of nuclear details of the cells with only the Bowman’s capsule still identifiable. Cases where the histology of the kidney was severely compromised were excluded from the study (3 out of 48 cases). The number of glomeruli were counted on 10 randomly chosen not overlapping microscopic fields at high power magnification (40×) referred to as “High Power Fields” (HPFs) using a standard light microscope (Nikon Eclipse E600 Tokyo, Japan) equipped with a microphotography system Nikon digital camera (DMX1200 Tokyo, Japan). Glomeruli were counted manually in each single HPF by two independent pathologists (IDA, GP) and the number was subsequently averaged per animal.

### 2.3. Immunohistochemical Examination

Expression of α-SMA antibody in samples was determined by immunohistochemistry [44]. In detail, all the paraffin-embedded kidney samples were cut into 4 μm thick sections and then mounted on positively charged glass slides (Bio-Optica, Milan, Italy); sections were deparaffined in xylene and in decreasing series of alcohol and peroxidases were blocked with a solution of hydrogen peroxide and methanol (4:1) for 15 min. Antigen retrieval pretreatments were executed using a heat-induced epitope retrieval (HIER) citrate buffer pH 6.0 (Bio-Optica, Milan, Italy) for 20 min at 98 °C. Further, immunohistochemistry was carried out following the protocol suggested by the MACH1 Universal HRP-Polymer Detection Kit (Cat. No: M1U539 G, L10, Bio-Optica, Milan, Italy). Sections were blocked with a protein block (MACH1, Biocare Medical LLC., Concord, CA, USA) for 30 min. Slides were incubated overnight at 4 °C with the primary antibody diluted in phosphate-buffered saline (PBS) (0.01 M PBS, pH 7.2). The primary antibody included monoclonal mouse anti-human smooth muscle actin, (1A4, DAKO) at 1:200 in PBS. Antibody deposition was visualized using the 3,3′-diaminobenzidine (DAB) chromogen diluted in the DAB substrate buffer; subsequently, the slides were counterstained with hematoxylin. Slides were washed two times (5 min each) in PBS between all incubation steps. In the corresponding negative control sections, the primary antibody was either omitted or replaced with a 1:20 dilution of rabbit serum (Code 011-000-120, Jackson Immuno Research, West Grove, PA, USA). Slides were examined and photographed with a light microscope (Nikon Eclipse E600 Tokyo, Japan) equipped with a microphotography system Nikon digital camera (DMX1200 Tokyo, Japan). For each assessed group, glomeruli were evaluated in 10 fields at 40× magnification by two independent pathologists (IDA, GP). The localization of α-SMA-positive cells was categorized as follows:

- Pattern 1, α-SMA-positive cells in the glomerulus;
- Pattern 2, periglomerular and glomerular α-SMA-positive cells;
- Pattern 3, periglomerular α-SMA-positive cells;
- Pattern 4, α-SMA-positive cells primary limited to blood vessel.

### 2.4. Statistical Analysis

The SPSS 20.0 package (SPSS Inc., Chicago, IL, USA) was used for statistical analysis of the data. The Mann–Whitney test, a nonparametric test, was used to assess differences in the number of glomeruli among evaluated groups and to investigate the localization of α-SMA-positive cells; *p*-values < 0.05 were considered statistically significant.

## 3. Results

### 3.1. Histological Examination

**Figure 1:**
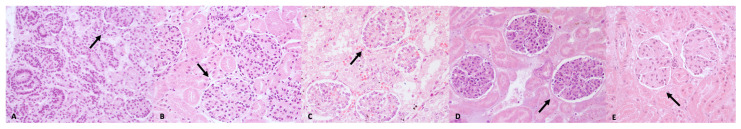
Morphometrical study of the kidney; examples of glomeruli (arrow) identified in histological sections of dogs from Group A (**A**), Group B (**B**), Group C (**C**), Group D (**D**), and Group E (**E**), respectively. Hematoxylin and Eosin (original magnification 40×). Mean values of the number of glomeruli were 9.48 ± 2.99 SD for animals in Group A, 5.6 ± 0.5 SD for animals in Group B, 4.4 ± 0.33 SD for animals in Group C, 3.6 ± 0.96 SD for animals in Group D, and 1.9 ± 0.45 SD for animals in Group E.

**Figure 2:**
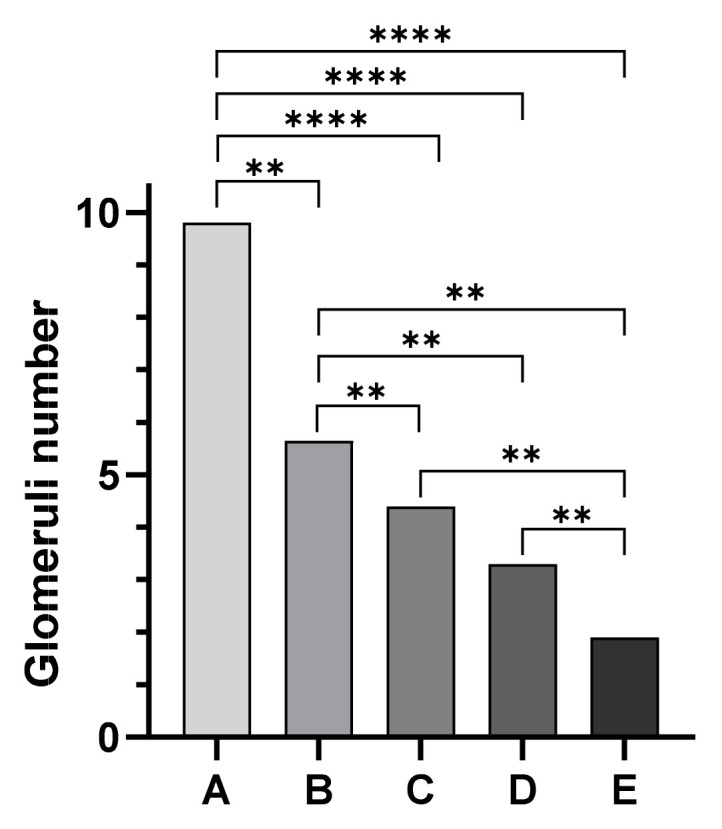
Statistical evaluation of the morphometrical study of the kidney: the Mann–Whitney test showed a statistically significant difference among assessed groups (*p* < 0.05) except between groups C and D. Group A vs. Group B ** *p* = 0.0050; Group B vs. Group C ** *p* = 0.0043; Group B vs. Group D ** *p* = 0.0023; Group B vs. Group E ** *p* = 0.0043; Group C vs. Group E ** *p* = 0.0043; Group D vs. Group E ** *p* = 0.0051; **** *p* < 0.0001.

Kidney histological sections were classified based on the degree of postmortem autolysis as follows: 25 cases exhibited Grade 1 (mild) postmortem changes, 14 cases showed Grade 2 (moderate) postmortem changes, and 6 cases showed Grade 3 (severe) postmortem changes. However, glomeruli were identifiable in all degrees (mild, moderate, severe) in postmortem autolysis. Mean values of the number of glomeruli were 9.48 ± 2.99 SD for animals in Group A, 5.6 ± 0.5 SD for animals in Group B, 4.4 ± 0.33 SD for animals in Group C, while Group D animals showed a mean number of glomeruli of 3.6 ± 0.96 SD. Finally, a mean number of 1.9 ± 0.45 SD glomeruli per field was observed in Group E (Figure 1). The Mann–Whitney test showed a statistically significant difference among assessed groups (*p* < 0.05) except between groups C and D (Figure 2).

### 3.2. Immunohistochemical Examination

**Figure 3:**
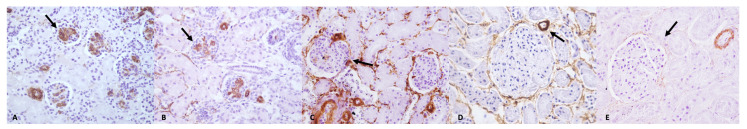
Immunohistochemical study of the kidney; examples of positivity to a-SMA (arrows) in histological kidney sections of dogs from Group A (A), Group B (B), Group C (C), Group D (D), and Group E (E) Hematoxylin and Eosin (original magnification 40×). Animals of Groups A and B showed α-SMA immunopositive cells in the glomerulus; in animals of Group C, α-SMA-positive cells were periglomerular and glomerular; animals of Group D showed mostly periglomerular α-SMA-positive cells while in animals of Group E, α-SMA-positive cells were primary limited to blood vessels.

**Figure 4:**
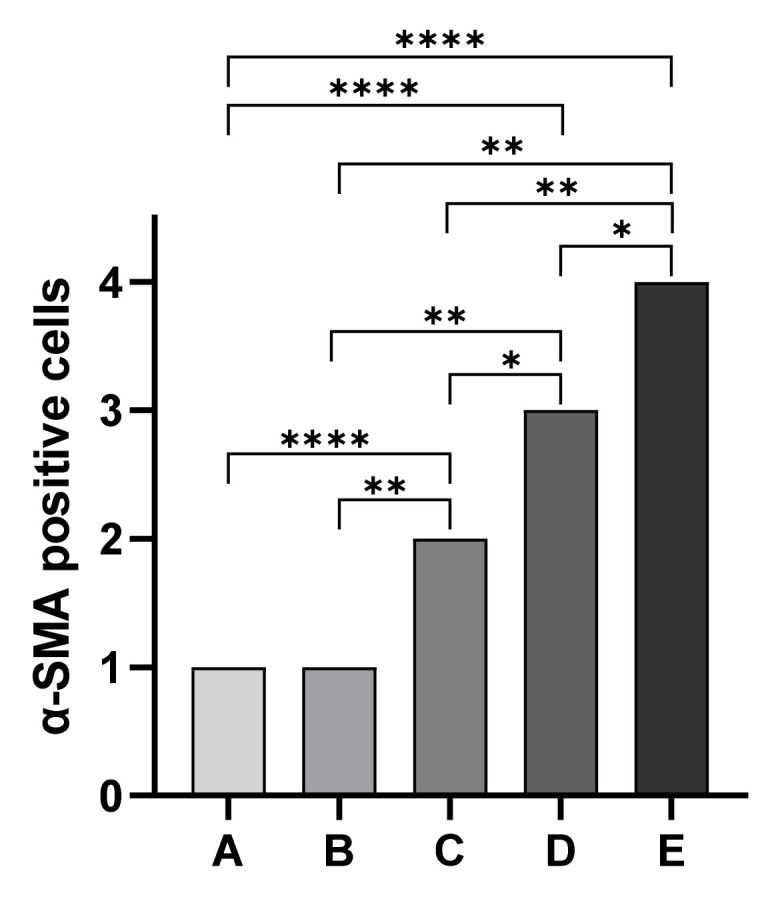
Statistical analysis of the localization of α-smooth muscle actin-positive cells. The Mann–Whitney test showed statistically significant differences in the localization of α-SMA-positive cells among groups except between Groups A and B * *p* = 0.0152; Group B vs. Group C ** *p* = 0.0022; Group B vs. Group D ** *p* = 0.0022; Group B vs. Group E ** *p* = 0.0022; Group C vs. Group E ** *p* = 0.0043; **** *p* < 0.0001.

Immunohistochemical staining for the evaluation of α-SMA antibody allowed us to observe differences in the localization of immunopositive cells among assessed groups. Overall, immunopositivity to α-SMA was observed in endothelial cells of blood vessels of all five assessed groups independently from the degree of tissue autolysis. In particular, kidney sections of cadavers of Group A showed strong α-SMA immunopositive cells in immature glomeruli in all assessed cases (20 out of 20 cases). Similarly, Group B kidney sections showed α-SMA immunopositive cells mainly localized in the mesangium in four out of six cases. All Group C kidney sections showed less immunopositivity of mesangium associated with α-SMA immunopositive cells localized in the vascular pole. In Group D animals, we observed a complete disappearance of immunopositivity to α-SMA in the glomerulus associated with α-SMA immunopositive cells localized in Bowman’s capsule. Finally, group E animals showed a complete disappearance of immunopositivity to α-SMA in the glomerulus (Figure 3). Statistical analysis showed statistically significant differences in the localization of α-SMA-positive cells among groups (*p* < 0.05) except between Groups A and B (Figure 4).

## 4. Discussion

Age estimation is an important topic in the forensic field, both in human and veterinary medicine; over the last decade, several studies have been published in both human and veterinary medicine regarding different methods of age estimation. Standardizing methods and procedures are essential both for cadavers and the livings, especially in puppies and children involved in forensic issues. Currently, there is no single method universally accepted; indeed, most of them are highly influenced by breed, sex, nutritional [45], hormonal, and pathological changes [45] and even from the preservation of the corpse or remains. In veterinary medicine, the most widely used method in living animals is the visual examination of dental eruption [18,24,39,46]. This is a non-invasive method that can be easily performed by veterinarians, but it is influenced by a wide range of factors, including diet, habits, and breed variability [23,39,46,47]. Another reliable method is the evaluation of the radiographic appearance of the ossification centers (OCs) in fore- and hind-limbs bones [35,36,38,48]. However, these studies are often performed on small populations and focus only on specific breeds [38,48]. References are not available for all breeds and estimation of the biological age of a dog can be challenging. Moreover, the development of the dog, from puppy to adult, is characterized by deep skeleton modifications that occur in a shorter period of time compared to humans. Consequently, no single method can be reliably used to accurately estimate the age of puppies. To overcome these limits, in our study, we investigated the changes in kidney development to propose a new scientific parameter to support the forensic veterinarians in correctly estimating the age of puppies. In particular, the morphometrical analysis of the kidney allowed us to observe a progressive reduction in the mean number of glomeruli per field with age. Furthermore, statistical analysis showed statistically significant differences among assessed groups except between groups C and D. The correlation between age of the puppies and the number of glomeruli could be explained by the progressive reduction in nephrogenic zone with age and the maturation of glomeruli. Indeed, the nephrogenic zone persists for approximately 15 days in dogs, but the growth and maturation of the kidney continues after nephrogenesis has ceased [42]. The similar number of glomeruli observed between groups C and D could be explained by the specific morphological development of the kidneys of puppies in the first weeks of life. Indeed, previously studies showed that the cortical zone is well-developed at 75 days of age and the histological features of the kidney are similar to those of adult dogs [42]. Regarding the immunohistochemistry evaluation, we observed statistically significant differences in α-SMA expression among assessed groups except between Groups A and B. Interestingly, immunopositivity to α-SMA was observed in endothelial cells of blood vessels in all assessed groups independently from the degree of tissue autolysis; these findings suggest a good preservation of α-SMA antigens in kidney tissue even in severe autolysis cases. Overall, our findings suggest α-SMA immunohistochemical changes during the first postnatal stage in puppy’s glomerulus. α-SMA expression changes observed in our study can be explained by postnatal puppies glomerular vasculogenesis. Indeed, it has been hypothesized that during glomerulogenesis there is a progressive and specific migration of extraglomerular endothelial cells and mesangial cells that rapidly differentiate into α-SMA-positive cells [49]. Our results are in agreement with previously published papers. Indeed, α-SMA is one of the six isoforms of the intra-cellular microfilament actin. Usually, the presence of α-SMA expression has been used as a marker for smooth muscle-derived tissue. However, it can also be expressed in non-muscle cells; in fact, α-SMA expression has been previously described in the kidney glomerulus in fetuses and infants [40] and in pericytes of the renal medulla [41]. Gonluses et al. demonstrated that mesangial cells in fetuses and infant kidneys are immunopositive to α-SMA, but they are negative in adult kidneys. Similarly, Carey et al. detected α-SMA immunopositive changes in rat kidneys during postnatal development. Our results are in accordance with previous studies and prove that α-SMA expression in dog kidneys ceases when glomerular development is complete [50].

## 5. Conclusions

Age estimation is a research topic that is particularly important, especially in correctly determining age in both the living and in cadavers. In veterinary forensic medicine, age estimation of puppies, both illegally imported in European countries and sold by breeders earlier than 8 weeks of age, is a topic of fundamental importance for public health reasons and for animal welfare. Therefore, the implementations of research in this field through guidelines, indications, or recommendations, at a national and international level, is essential in order to standardize the procedure of age estimation in the different European states. Our findings suggest a potential use of kidney morphometrical and immunohistochemical examinations together as an indirect parameter to assess the age of puppies in veterinary forensic pathology in association with other methods described in the literature. Overall, our data increase the knowledge available regarding age estimation methods; however, further studies will be needed in order to exclude or detect differences in glomeruli maturation times in dogs of different breeds and to identify additional immunohistochemical markers to better estimate the age of puppies in veterinary forensic pathology.

## Data Availability

The data presented in this study are available on request from the corresponding author.
